# Genosensing Applications of Glassy Carbon Electrodes Modified with Multi-Walled Carbon Nanotubes Non-Covalently Functionalized with Polyarginine

**DOI:** 10.3390/mi13111978

**Published:** 2022-11-15

**Authors:** Pablo Gallay, Michael López Mujica, Soledad Bollo, Gustavo Rivas

**Affiliations:** 1 INFIQC, Departamento de FIsicoquímica, Facultad de Ciencias Químicas, Universidad Nacional de Córdoba, Ciudad Universitaria, Córdoba 5000, Argentina; 2 Centro de Investigación de Procesos Redox, CIPRex, Facultad de Ciencias Químicas y Farmacéuticas, Universidad de Chile, Sergio Livingstone 1007, Independencia, Santiago 8380000, Chile; 3 Departamento de Química Farmacológica y Toxicológica, Facultad de Ciencias Químicas y Farmacéuticas, Universidad de Chile, Sergio Livingstone 1007, Independencia, Santiago 8380000, Chile

**Keywords:** carbon nanotubes, impedimetric biosensor, DNA adsorption, polyarginine, nanotechnology, microRNA-21

## Abstract

We report the advantages of glassy carbon electrodes (GCE) modified with multi-walled carbon nanotubes (MWCNTs) non-covalently functionalized with polyarginine (PolyArg) for the adsorption and electrooxidation of different DNAs and the analytical applications of the resulting platform. The presence of the carbon nanostructures, and mainly the charge of the PolyArg that supports them, facilitates the adsorption of calf-thymus and salmon sperm double-stranded DNAs and produces an important decrease in the overvoltages for the oxidation of guanine and adenine residues and a significant enhancement in the associated currents. As a proof-of-concept of possible GCE/MWCNTs-PolyArg biosensing applications, we develop an impedimetric genosensor for the quantification of microRNA-21 at femtomolar levels, using GCE/MWCNTs-PolyArg as a platform for immobilizing the DNA probe, with a detection limit of 3fM, a sensitivity of 1.544 × 10^3^ Ω M^−1^, and a successful application in enriched biological fluids.

## 1. Introduction

Electrochemical (bio)sensors have emerged as important analytical tools for the quantification of analytes of clinical, pharmaceutical, environmental, and foods safety relevance [1,2,3], and currently there is an increasing interest in the development of new biosensing platforms and new transduction schemes. The incorporation of carbon nanomaterials has largely improved the analytical performance of the resulting bioanalytical platforms because of their unique physical and chemical properties [4,5,6,7,8]. Among these carbon nanostructures, carbon nanotubes (CNTs) have been widely used for the construction of electrochemical sensors [9,10,11]. 

To make possible use of CNTs for the development of electrochemical sensors, different functionalization routes have been proposed [12,13,14]. We have reported the successful functionalization of CNTs with polymers including polyethylenimine [15], polylysine [16], and polyhistidine [17]; proteins such as glucose oxidase [18], cytochrome C [19], avidin [20,21,22], and concanavalin [23]; and nucleic acids, namely, calf-thymus double-stranded DNA [24]. We have also reported the successful non-covalent functionalization of MWCNTs with polyarginine (PolyArg) [25] and the sensing applications for the quantification of uric acid [25] and ethanol [26]. Jalit et al. [27] demonstrated the advantages of using GCE modified with MWCNTs dispersed in polylysine for the electrooxidation of oligo and polynucleotides, and reported a model hybridization sensor using oligo(dC)_11_ as probe and oligo(dG)_11_ as target. 

The goal of this work is to evaluate the adsorption and electrooxidation of different DNAs at glassy carbon electrodes (GCE) modified with MWCNTs non-covalently functionalized with PolyArg and discuss, as a proof-of-concept, the analytical usefulness of the resulting GCE/MWCNTs-PolyArg to build an impedimetric genosensor for miRNA-21 (miRNA-21) through the use of MWCNTS-PolyArg as a platform for anchoring the DNA probe. DNA-based nanostructured sensors combine the advantages of highly advanced nanomaterials and DNA receptors in one architecture. In the following sections, we discuss the electrochemical response of salmon sperm and calf-thymus double-stranded DNAs at GCE/MWCNTs-PolyArg as well as the optimization of the miRNA-21 biorecognition layer and the analytical performance of the resulting genosensor. 

## 2. Materials and Methods

### 2.1. Reagents and Materials

Double-stranded calf-thymus DNA (dsDNA_CT_, catalog number D-4522), salmon sperm double-stranded DNA (dsDNA_S_), poly-L-arginine (PolyArg, mol wt > 70,000), hydroquinone (HQ), and benzoquinone (Bz) were purchased from Sigma-Aldrich (USA). Multi-walled carbon nanotubes powder (MWCNTs) (30 ± 15 nm diameter, 1–5 microns length) was obtained from NanoLab (USA). Ethanol was purchased from J. T. Baker. Other chemicals were reagent grade and used without further purification. The oligonucleotides were purchased from Life Technology (USA) and the sequences are the following:

oligo_21_: 5′-TCA-ACA-TCA-GTCTGA-TAA-GCT-A-3′
microRNA-21: 5′-UAG-CUU-AUC-AGA-CUG-AUGUUG-A-3′
fully non-complementary sequence: 5′-GGG-GGG-GGGGGG-GGG-3′
single-base mismatch: 5′-UAG-CUU-AUC-ACA-CUGAUG-UUG-A-3′

Ultrapure water (ρ = 18.2 MΩ cm) from a Millipore Milli-Q system was used for pre paring all the solutions.

### 2.2. Apparatus

Electrochemical measurements were performed with an Epsilon (BAS) potentiostat. Glassy carbon electrodes (GCE, 3 mm diameter, CHI), either bare or modified with MWCNTs functionalized with PolyArg, were used as working electrodes. As counter and reference electrodes, we used a platinum wire and Ag/AgCl, 3M NaCl, respectively. All potentials refer to the latter.

The sonication of the MWCNTs-PolyArg mixture was conducted with an ultrasonic processor (VCX 130W, Sonics and Materials, Inc., Newtown, CT, USA), of 20 kHz frequency with a titanium alloy microtip. An Allegra^TM^ 21 ultracentrifuge (Beckman Coulter) with a F2402H rotor was used to centrifuge the samples after sonication. 

Electrochemical impedance spectroscopy (EIS) experiments were performed in a 1.0 × 10^−3^ M HQ/Bz solution (prepared in 0.050 M phosphate buffer solution pH 7.40) by applying a perturbation of sinusoidal potential of 10 mV in the frequency range of 10^5^–10^−1^ Hz and a working potential of 0.200V. The spectra were analyzed and fitted using the Z-view program.

The spectrophotometric experiments were performed with a Shimadzu UV 1700 Pharma spectrometer. 

### 2.3. Preparation of MWCNTs Functionalized with PolyArg (MWCNTs-PolyArg)

**Figure 1:**
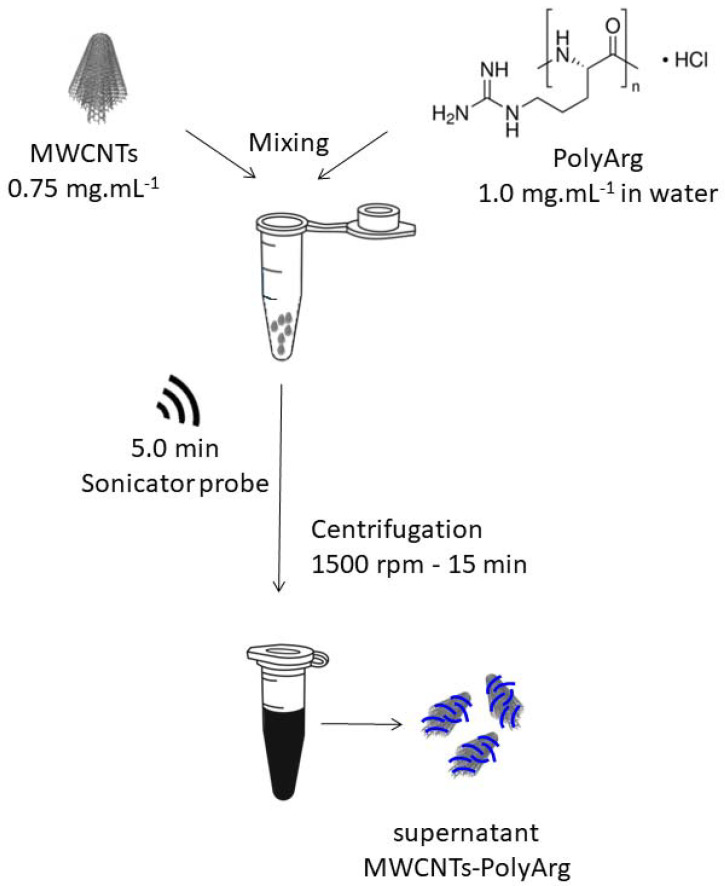
Schematic representation of the MWCNTs’ functionalization with PolyArg.

The dispersion of MWCNTs with PolyArg was prepared by sonicating 0.75 mg of MWCNTs with 1.0 mL of PolyArg solution (1.0 mg mL^−1^, prepared in water) with a sonicator probe (amplitude 50%) for 5.0 min. The amplitude was 50% and the mixture was kept in an ice bath during the procedure. After this treatment, the samples were centrifuged at 1500 rpm for 15 min and the supernatant collected for further work (See scheme shown in Figure 1). 

### 2.4. Preparation of GCE Modified with MWCNT-PolyArg (GCE/MWCNT-PolyArg)

GCEs were first polished with alumina slurries of 1.0, 0.3, and 0.05 μm for 1.0 min each, rinsed carefully with water, sonicated for 10 s in water/ethanol 50:50 *v*/*v*, and dried under a nitrogen stream. GCE/MWCNT-PolyArg was obtained by dropping 10 µL of MWCNT-PolyArg onto the glassy carbon surface and dried at room temperature. 

### 2.5. Preparation of GCE/MWCNTs-PolyArg Modified with DNA 

The DNAs were adsorbed by dropping at GCE or GCE/MWCNT-PolyArg 10 µL of the corresponding solution prepared in acetate buffer solution (0.200 M, pH 5.00). Once the solvent was evaporated, the electrodes were washed by immersion in the acetate buffer solution for 5 s and then transferred to the stripping solution. The determination was performed by Adsorptive Stripping Linear Scan Voltammetry (AdSLSV) in a 0.200 M acetate buffer solution pH 5.00 at 0.050 V s^−1^. The analytical signals were obtained after subtracting the background currents.

### 2.6. microRNA-21 Genosensor 

**Figure 2:**
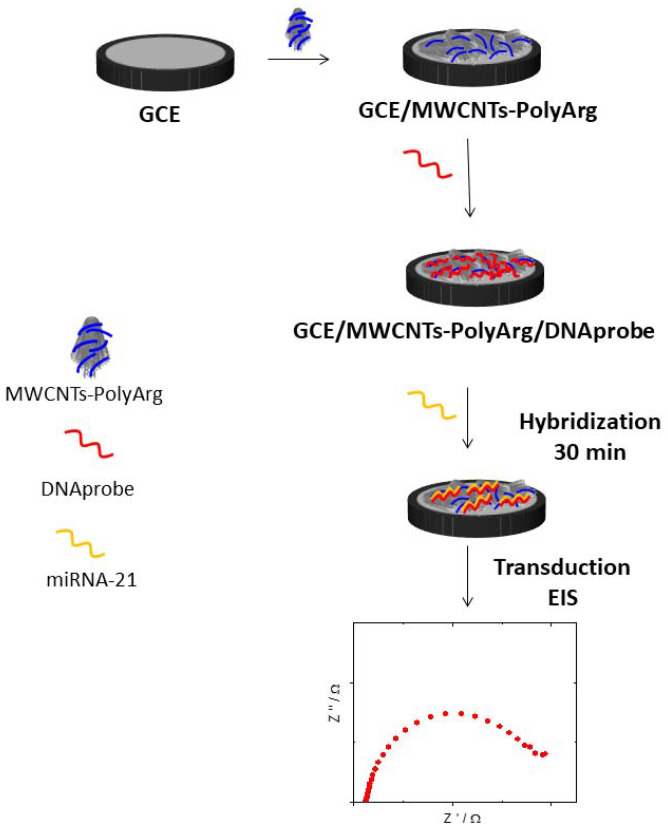
Schematic representation of the genosensor preparation.

The DNA probe (oligo_21_, DNAp) was immobilized at GCE/MWCNT-PolyArg according to the protocol previously indicated in Section 2.5. The electrode was washed by immersion in a 0.050 M phosphate buffer solution + 0.200 M NaCl for 10 s and was then allowed to hybridize by dropping an aliquot of 20 µL microRNA-21 solution for 30 min. After washing with the phosphate buffer solution, the transduction was performed by EIS in a 1.0 × 10^−3^ M HQ/Bz solution (See scheme displayed in Figure 2).

## 3. Results 

### 3.1. Adsorption and Electrooxidation of Different DNAs at GCE/MWCNT-PolyArg

**Figure 3:**
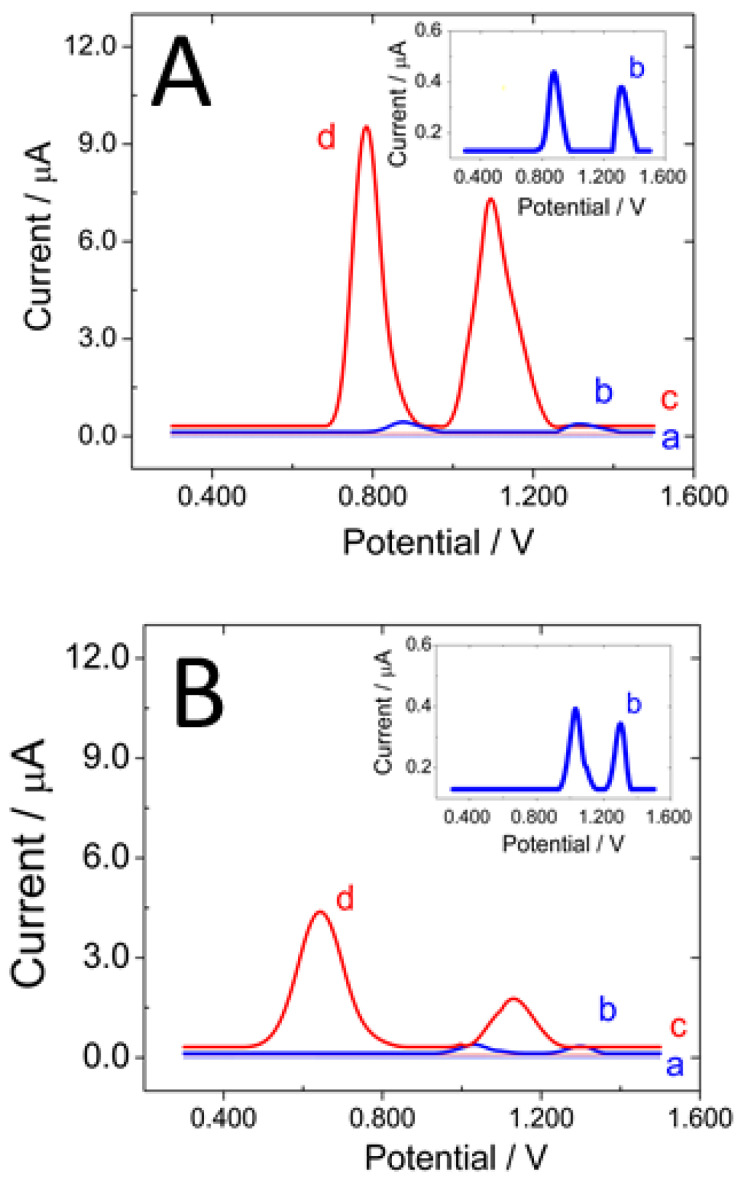
(**A**) Linear scan voltammograms obtained after baseline correction for: GCE (a), GCE/ds-DNAS (b), GCE/MWCNTs-PolyArg (c), and GCE/MWCNTs-PolyArg/ds-DNAS (d). (**B**) Linear scan voltammograms obtained after baseline correction for: GCE (a), GCE/ds-DNACT (b), GCE/MWCNTs-PolyArg (c), and GCE/MWCNTs-PolyArg/ds-DNACF (d). Supporting electrolyte: 0.200 M acetate buffer solution pH 5.00; dsDNAs accumulation: by deposition and solvent evaporation until dryness; dsDNAs Concentration: 100 ppm; scan rate: 0.050 V s^−1^.

Figure 3A displays linear scan voltammograms (LSV) obtained in a 0.200 M acetate buffer solution pH 5.00 before (a,c) and after (b,d) the deposition of 100 ppm dsDNA_s_ at bare GCE (a,b) and GCE/MWCNTs-PolyArg (c,d). At GCE, there are two oxidation peaks, at 0.86 V and 1.31 V, due to the oxidation of guanine and adenine residues [28,29]. The LSV obtained at GCE/MWCNTs-PolyArg shows two oxidation peaks at potentials 93 and 223 mV more negative with currents 32.5 and 26.9 times higher than those obtained at bare GCE, for guanine and adenine, respectively. This decrease in the overvoltages and the enhancement in the oxidation currents are clear evidence of the facilitated interaction of the nucleic acid with the walls of the MWCNTs and the positively charged polycation that wraps them. It is important to mention that the oxidation of 5.0 × 10^−4^ M guanine and adenine at GCE/MWCNTs-PolyArg occurs at 0.64 V and 1.07 V, respectively (not shown). Figure 3B depicts the voltammetric response obtained in a 0.200 M acetate buffer solution pH 5.00 before (a,c) and after (b,d) the deposition of 100ppm dsDNA_CT_ at bare GCE (a,b) and at GCE/MWCNTs-PolyArg (c,d). In the case of this polynucleotide, the presence of MWCNTs-PolyArg produced a very important decrease in the overvoltages for the oxidation of guanine (E_p_ = 0.640 V versus 1.100 V for GCE/MWCNTs-PolyArg and GCE, respectively) and adenine (E_p_ = 1.120 V versus 1.300 V for GCE/MWCNTs-PolyArg and GCE, respectively), due to the considerably higher number of base-pairs of dsDNA_CT_ and, consequently, the higher negative charge density of the sugar–phosphate backbone. Compared to GCE, there is a significant enhancement of the associated currents at a factor of 16.7 and 6.5 for guanine and adenine, respectively, although it is smaller than the one observed for dsDNA_S_. This less pronounced enhancement in the currents is due to the larger size of dsDNA_CT_ that makes the accessibility for the electrooxidation of guanine and adenine residues more difficult. In fact, the currents are the result of a compromise between the accessibility of the guanine/adenine sites to the carbon surface, the surface packing, and the orientation of the polynucleotide [30,31,32]. The results discussed above demonstrate the relevance of the PolyArg that supports the MWCNTs, not only to disperse them but also to make more favorable the interaction of DNAs at the electrode surface.

### 3.2. GCE/MWCNT-PolyArg as a Platform to Build a miRNA-21 Genosensor

**Figure 4:**
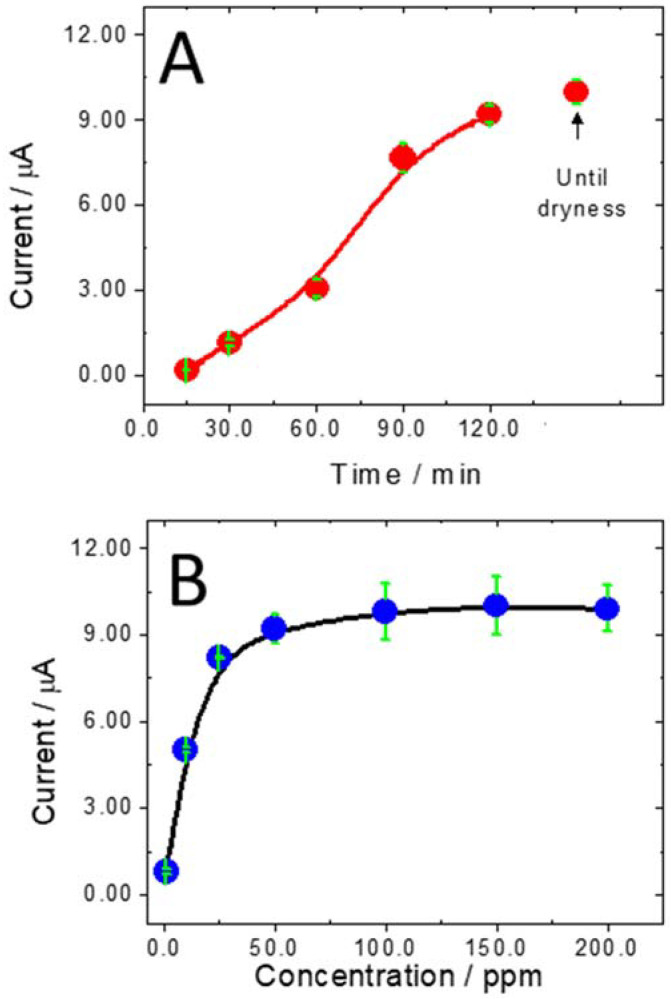
(**A**) Influence of the accumulation time of oligo_21_ at GCE/MWCNTs-PolyArg on the guanine oxidation peak current. Oligo_21_ concentration: 100 ppm. (**B**) Effect of oligo_21_ concentration deposited at GCE/MWCNTs-PolyArg on the guanine oxidation peak current. Accumulation time: until dryness. Other conditions are as in Figure 3.

As proof-of-concept of the biosensing usefulness of MWCNTs-PolyArg nanohybrid, we develop an impedimetric biosensor for the quantification of miRNA-21. The biorecognition layer of the genosensor was obtained by adsorption of oligo_21_ at GCE/MWCNTs-PolyArg as probe. Figure 4 shows the effect of the adsorption time of oligo_21_ at GCE/MWCNTs-PolyArg on the guanine oxidation current. As the adsorption time increases, there is an increment in the oxidation signals, which starts to level off at 100 min, indicating a saturation of the available sites for the adsorption of the nucleic acid. For comparison, the guanine oxidation signal obtained after deposition of 100 ppm oligo_21_ at GCE/MWCNTs-PolyArg and solvent evaporation until dryness is also included. This current shows no significant difference with the one obtained after 2 h adsorption. Figure 4B displays the guanine oxidation currents for increasing concentrations of oligo_21_ deposited at GCE/MWCNTs-PolyArg. The currents almost level off for concentrations higher than 50 ppm. Therefore, the selected conditions to ensure the full coverage of the surface were the deposition of an aliquot of 100 ppm oligo_21_ solution at GCE/MWCNTs-PolyArg surface followed by the evaporation of the solvent. 

**Figure 5:**
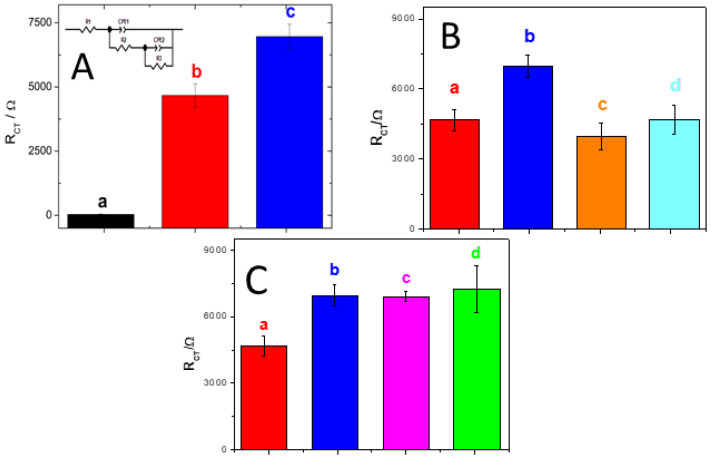
(**A**) Bar plot for the R_ct_ of the redox marker obtained after the different steps during the construction of the genosensors and subsequent hybridization: GCE/MWCNTs-PolyArg (a), GCE/MWCNTs-PolyArg/oligo_21_ (b), and GCE/MWCNTs-PolyArg/oligo_21_ in the presence of 1.0 × 10^−13^ M miRNA-21 (c). The inset shows the corresponding equivalent circuit. (**B**) Bar plots for R_ct_ of the redox marker obtained at: (a) GCE/MWCNTs-PolyArg/oligo_21_ in the absence (a) and in the presence of 1.0 × 10^−13^ M miRNA-21 (b); 1.0 × 10^−13^ M fully non-complementary sequence (c); 1.0 × 10^−13^ M one-base mismatch sequence (d). (**C**) Bar plots for R_ct_ of the redox marker obtained at GCE/MWCNTs-PolyArg/oligo_21_ in the absence (a) and presence of 1.0 × 10^−13^ M miRNA-21 (b); serum 1/1000 enriched with 1.0 × 10^−13^ M miRNA.21 (c); urine diluted 1/10 enriched with 1.0 × 10^−13^ M miRNA-21 (d). Redox marker: 1.0 × 10^−3^ M HQ/Bz solution; EIS parameters: amplitude, 0.010 V, frequency range, 1.0 × 10^−2^ to 1.0 × 10^6^ Hz, working potential, 0.200 V.

Figure 5A displays a bars plot for the charge transfer resistance (R_ct_) obtained from Nyquist plots after each step during the building of the impedimetric biosensor: (a) GCE/MWCNTs-PolyArg, (b) GCE/MWCNTs-PolyArg/oligo_21_, and (c) GCE/MWCNTs-PolyArg/oligo_21_ after hybridization in the presence of 0.10 nM miRNA-21. The experimental results were fitted with a Randles circuit (inset), which involves the solution resistance (R_s_), the redox marker charge transfer resistance (R_ct_), the double-layer capacitance (C_dl_), and the impedance of Warburg (W). Once the oligo_21_ (DNAp) is immobilized at GCE/MWCNTs-PolyArg, there is an important increment in the R_ct_ (40 vs. 4700 Ω) due to the blockage effect of the non-conductive DNA layer. After hybridization with 0.10 nM miRNA-21, there is a new increment of the R_ct_ (7000 Ω) as a consequence of the additional blockage produced by the double helix formation. 

The calibration plot presented a linear relationship between 10^−14^ M and 10^−12^ M miRNA-21, with a sensitivity of 1.544 × 10^3^ Ω M^−1^ (r^2^ = 0.992), a detection limit (DL) of 3.3 fM, and a quantification limit (QL) of 10 fM (calculated according to 3σ/S and 10σ/S, for DL and QL, respectively, where S is the sensitivity and σ is the standard deviation of the blank signal). The reproducibility for 1.0 × 10^−13^ M target using three electrodes prepared with the same MWCNTs-PolyArg/oligo_21_ nanoplatform was 5.7%. 

Figure 5B displays the bars plot for the R_ct_ of the redox marker obtained at GCE/MWCNTs-PolyArg/oligo_21_ nanoplatform in the absence (a) and presence of: (b) 1.0 × 10^−13^ M of miRNA-21, (c) a fully non-complementary sequence, and (d) one-base mismatch sequence. Almost no changes were observed in the presence of the fully non-complementary and one-base mismatch sequences, clearly indicating the selectivity of the miRNA-21 genosensing.

We also explored the potential practical application of the biosensor to detect miRNA-21 in real samples such as urine and reconstituted human serum samples. Figure 5C displays the bars plot for the R_ct_ obtained at GCE/MWCNTs-PolyArg/oligo_21_ (a) and at GCE/MWCNTs-PolyArg/oligo_21_ in the presence of: miRNA-21 1.0 × 10^−13^ M (b) and samples of human serum (c) and urine (d) diluted 1/10 with 0.050 M phosphate buffer solution pH 7.40 and enriched with 1.0 × 10^−13^ M miRNA-21. Excellent recovery percentages were obtained in both cases (91.0% and 94.0% for blood serum and urine, respectively), demonstrating the potential application of the proposed genosensor for the quantification of miRNA-21 in different biological fluids.

A comparison of the analytical performance of our biosensor with the most relevant miRNA-21 electrochemical biosensors reported in the past couple of years is displayed in Table 1. Even when there are some amplified schemes that present lower detection limits than our sensor, it represents an interesting strategy for quantifying miRNA-21 at fM levels in a non-amplified, label-free, fast, and friendly way [33,34,35,36,37,38,39,40,41,42,43,44,45,46,47,48,49]. 

## 4. Conclusions

We report the advantages of MWCNTs-PolyArg nanohybrid for the adsorption and electrooxidation of calf-thymus and salmon sperm double-stranded DNAs. Their most favorable interaction with the aromatic rings of MWCNTs and the positive charge of the polycation that wraps the nanostructures produces a significant decrease in the overvoltages for the oxidation of guanine and adenine residues as well as an important enhancement in the associated currents. This favorable interaction of DNAs with the nanobioplatform allowed us to use MWCNTs-PolyArg as a building block for the construction of a simple, easy-to-prepare, friendly, label- and amplification-free impedimetric genosensor for the highly selective and sensitive quantification of miRNA-21 at femtomolar levels in a very competitive way. 

## Figures and Tables

**Table 1:** Comparative table of the analytical characteristics of the most relevant miRNA electrochemical biosensors reported in the past couple of years.

Electrochemical Impedance Spectroscopy—EIS					
**Target**	**Platform-Analytical Signal**	**LOD**	**Linear Range R^2^**	**Real Sample**	**Ref**
miRNA-21	Polypyrrole modified pencil graphite electrode. Change in Rct or meldola blue signal.	12.25 nM	-/0.977	MCF-7 and HUH-7	[33]
miRNA-21	AuNPs-decorated MoS_2_ nanosheet (AuNPs@MoS_2_) as electrode modifier and signal-amplifier element.	7.08 fM	10 fM–1.0 nM/0.99	Human serum	[34]
miRNA-21	Hybridization chain reaction (HCR) amplification-based impedimetric biosensor. Two sequences are used to trigger HCR amplification (H1 and H2).	4.63 fM	10 fM–50 pM/0.998	Human serum	[35]
miRNA-21	Impedimetric detection of the miRNAs by the signal amplification of insulating biomaterials. Biotinylated miRNA with the same sequence as that of target miRNA was captured by the sensor.	0.1 fM	0.1–250 fM/-	Cell lysates	[36]
miRNA-34a	Electrochemical entrapment of the probe (antimiRNA-34a) into polypyrrole (PPy) performed by electropolymerization.	0.2 μg.mL^−1^	5–80 μg.mL^−1^/0.986	MCF-7	[37]
miRNA-21	GCE/MWCNTs-PolyArg/DNAp. Rct of the redox probe hydroquinone/benzoquinone as analytical signal.	3 fM	1.0 × 10^−14^–1.0 × 10^−12^ M 0.992	Serum and urine	This Work
**Amperometry—** **Differential Pulse Voltammetry (DPV)**					
miRNA-21	Thiolated DNA capture probe immobilized at gold nanoparticles–nanostructured electrode surface. Analytical signal obtained by amperometry using a specific antibody, horseradish peroxidase/H_2_O_2_/hydroquinone.	29 fM	0.096−25 pM/-	Human serum	[38]
miRNA-21	Competitive DNA-target miRNA hybridization on the surface of magnetic microbeads. Amperometric transduction at screen-printed carbon electrodes.	0.2 nM	0.7–10.0 nM/0.999	MCF-7 and MCF-10A	[39]
miRNA-21	Capture DNA (cDNA) self-assembled on the surface of gold electrode. Analytical signal obtained from the DPV current due to the accumulation of methylene blue.	0.01 fM	0.05–5 fM/0.995	Human serum	[40]
miRNA-21	Covalent assembling of the capture DNA at the gold nanoparticle-coated glassy carbon electrode. Analytical signal due to DPV current of the accumulated methylene blue at the hybrid obtained by sandwich hybridization with a long guanine-rich sequence.	56 fM	0.5–80 pM/0.991	Medulloblastoma cell extracts and clinical CSF	[41]
miRNA-21	Carboxylated single-walled carbon nanotubes immobilized at an aryldiazonium salt-modified electrode, as a platform to attach a ferrocene-labeled single-stranded DNA by non-covalent adsorption. Electrochemical signal due to the release of this labeled DNA.	3.5 fM	0.01 pM–100 pM/0.996	-	[42]
miRNA-21/155/A-205/let-7b	miRNA captured from lysed exosomes in specially designed capture probe modified magnetic beads, followed by T4 DNA polymerase-mediated and in situ formation of chimeric 5′ -miRNA-DNA-3′ (target).	92 aM	100 aM–10 pM	Human serum	[43]
miRNA-21	A nanocomposite containing thionine, reduced graphene oxide, ordered mesoporous carbon, and gold nanoparticles was used to increase the specific surface area of a glassy carbon electrode and amplify the DPV signal.	0.046 fM	0.1 fM–1.0 pM	Human serum	[44]
**Surface Plasmon Resonance (*SPR*)**					
miRNA-21	Platform obtained by self-assembling of two poly(diallyldimethylammonium chloride) (PDDA) bilayers and graphene oxide at a gold surface modified with 3-mercaptopropane sulfonate (MPS), followed by the covalent attachment of the DNA probe.	0.3 fM	1.0 × 10^−15^–1.0 × 10^−6^ M	Urine	[45]
miRNA-21/miRNA-155	Sensor based on two-dimensional nanomaterial of antimonene for the specific label-free detection of miRNA-21 and miRNA-155.	10 aM	10^−17^ to 10^−11^ M	-	[46]
miRNA	Enzyme-free amplified biosensor based on gold nanoparticles coupled with DNA supersandwich. The DNA-linked gold nanoparticles as the primary amplification element hybridizes with the capture DNA on the Au film and initiates the subsequent secondary amplification. In the presence of target, stem-loop structure of capture DNA on the Au film surface was unfolded and DNA-linked gold nanoparticles were bound to Au film by hybridization with terminus of capture DNA.	8 fM	-	Human serum	[47]
miRNA	Based on the produced-I_2_ triggered chemical etching of gold nanorods to a smaller size, resulting in a significant blue shift and high decrease of the localized surface plasmon resonance (LSPR) scattering	71.22 fM	0.1–10.000 pM/0.995	Human serum	[48]
miRNA-141	Based on two layers of graphene oxide–gold nanoparticles (GO–AuNPs).	0.1 fM	-	Human serum	[49]

## Data Availability

Not applicable.
